# Evaluation of NSE and S100B in patients with tick‐borne encephalitis

**DOI:** 10.1002/brb3.1160

**Published:** 2018-11-22

**Authors:** Piotr Czupryna, Sambor Grygorczuk, Sławomir Pancewicz, Renata Świerzbińska, Joanna Zajkowska, Katarzyna Krawczuk, Justyna Dunaj, Justyna Filipiuk, Ewelina Kruszewska, Karol Borawski, Anna Moniuszko‐Malinowska

**Affiliations:** ^1^ Department of Infectious Diseases and Neuroinfections Medical University of Białystok Białystok Poland; ^2^ Department of Paediatric Infectious Diseases Medical University of Białystok Białystok Poland; ^3^ Regional Center of Blood Donation Hajnowka Poland

**Keywords:** neurodegeneration, NSE, S100B, TBE

## Abstract

**Introduction:**

The aim of this study was the assessment of neuron‐specific enolase (NSE) and S‐100 concentration in serum and cerebrospinal fluid (CSF) in patients with different clinical forms of tick‐borne encephalitis (TBE).

**Material and Methods:**

The serum and CFS concentrations of S100B and NSE of 43 patients with TBE were measured with ELISA method using commercial kits: NSE and S100B Elisa Kit (DRG, Germany). Subjects were divided into: Group I—patients with meningoencephalitis (*n* = 17) and Group II—patients with meningitis (*n* = 26). None of the patients reported any neurodegenerative disorder that could affect the results of the study. The control group (CG) consisted of 13 patients. These patients were admitted to the hospital because of headache, and the CSF examination excluded inflammatory process. Samples were collected on admission (sample 1) and after treatment (sample 2).

**Results:**

Neuron‐specific enolase concentration in CSF was higher in group I than in group II (*p* = 0.0002) and controls (*p* = 0.04). NSE concentration was higher in the second serum and CSF sample in both groups. S100B concentration did not differ between TBE patients and controls. NSE concentration in serum after 14 days was higher in the sequelae group (34.3 ± 9.7 vs. 16.7 ± 15, *p* = 0.04). Also, NSE serum sample 2/serum sample 1 ratio was significantly higher in the sequelae group (3.57 ± 0.92 vs. 1.53 ± 1.99, *p* = 0.04). Receiver Operating Characteristic curve analysis indicated that NSE concentration in serum II differentiates sequelae group from other meningoencephalitis patients (*p* = 0.0001). S100B serum sample 2/CSF sample 2 ratio was lower in the sequelae group (0.05 ± 0.1 vs. 0.37 ± 0.28, *p* = 0.02).

**Conclusions:**

(a) Neurodegeneration process is present in TBE encephalitis. (b) NSE concentration correlates with inflammatory parameters in CSF in TBE. (c) Neurodegeneration is present even after clinical recovery of TBE. (d) NSE could be used in the prediction of TBE course. (e) S‐100 did not differ between TBE patients and controls.

## 1 INTRODUCTION

Tick‐borne encephalitis (TBE) is an infectious disease of the central nervous system (CNS) caused by tick‐borne encephalitis virus (TBEV) of *Flavivirus* genus, transmitted by *Ixodes* ticks. It is endemic in the temperate zone of Asia, Eastern and Central Europe, where several thousand cases are reported annually. In Poland, between 200 and 300 are noted per year. The majority of cases is noted in the Podlaskie Province (www.pzh.gov. pl).

Tick‐borne encephalitis may present with different clinical manifestations from asymptomatic to life‐threatening and/or causing permanent neurological and cognitive deficits. We can differentiate three distinct clinical forms of TBE: meningitis (fever, headaches, vomits, nausea, and neck stiffness), meningoencephalitis (consciousness disturbances, focal neurological symptoms in addition to clinical findings of meningitis), meningoencephalomyelitis: flaccid mono‐, para‐ or tetraparesis in addition to clinical findings of meningoencephalitis (Bogovic & Strle, 2015; Czupryna et al., 2011).

The severity correlates with age and clinical conditions causing immunosuppression, but the variability cannot be explained by these obvious factors exclusively (Bogovic & Strle, 2015).

The CNS tissue damage typical for severe clinical manifestations of TBE may result from the TBEV cytopathic effect and the local inflammatory response (Gelpi et al., 2006). TBEV RNA is detectable in cerebrospinal fluid (CSF) only in the earliest stage of meningitis and correlates with a prolonged host response to a relatively low or transient virus load and a possible participation of immune‐driven pathology (Saksida et al., 2005). The animal models show that the outcome of the neurotropic *Flavivirus* challenge may stem from the phenomena occurring at several stages of the infection: the control of the primary focus of the infection (virus spread with seroconversion and possible symptomatic disease vs. quick suppression of the infection by the local innate responses), the extent of the peripheral infection, and related inflammatory response, ability of the virus to spread into CNS (neuroinvasiveness affecting the progression from an unspecific febrile disease to meningitis/meningoencephalitis), and the neurovirulence during neurological phase (Palus et al., 2013; Tun et al., 2014). The last depends on both virus cytopathic effect on neurons and on secondary immunopathology related to host response and determines the difference between the uncomplicated meningitis and severe neurological forms of the disease. These observations suggest that complex and individually variable pathology may determine TBE manifestations in humans as well.

Many TBE patients develop neurological and psychiatric sequelae. So‐called post‐encephalitic TBE syndrome was described in 35%–58% of patients. It may cause long‐term morbidity that often affects the patient's quality of life and forces changes in a lifestyle. The most common reported symptoms were cognitive or neuropsychiatric complaints (reduced stress tolerance, impaired ability to memorize), balance disorders, headache, dysphasia, hearing defects, and limb paresis (Haglund & Gunther, 2003; Kaiser, 1999, 2008; Karelis et al., 2012; Laursen & Knudsen, 2003).

According to the guidelines, there is no specific treatment for TBE, yet symptomatic treatment should be introduced when indicated: antipyretics and analgesics, antiemetics, therapies for epileptic seizures and cerebral edema, respiratory and circulatory support, control of electrolyte and fluid balance, and treating neurological and systemic complications to prevent secondary neuronal damage (Taba et al., 2017).

The pathogenesis of different clinical presentations and sequelae development in TBE has not been fully recognized so far. Neurotropic viruses can induce significant neuronal dysfunction and degeneration of specific neuronal populations, sometimes leading to devastating or life‐threatening consequences to the host (Amor, Puentes, Baker, & Valk, 2010). TBE virus neurotropism preferentially targets large neurons of anterior horns, medulla oblongata, pons, dentate nucleus, Purkinje cells, and striatum. It was confirmed that the disease may lead to nerve cell destruction (Gelpi et al., 2005; Ludlow et al., 2016).

Therefore, in our study, we decided to assess the concentrations of S100B and neuron‐specific enolase (NSE) as proven to be potential biomarkers of CNS damage as well as good predictors of mortality and sequelae development in many diseases (Fowler, Ygberg, Bogdanovic, & Wickström, 2016; Hajduková et al., 2015).

### 1.1 Aim

The aim of this study was the assessment of NSE and S‐100 concentration in serum and cerebrospinal fluid (CSF) in patients with different clinical forms of TBE: meningitis and meningoencephalitis.

Detailed aims:

1. Evaluation of S100B protein and NSE in patients with TBE in serum and cerebrospinal fluid before and after treatment.
2. Comparison of S100B protein and NSE concentrations in serum and cerebrospinal fluid in patients with the inflammation of CNS with patients without CNS involvement.
3. Comparison of S100B protein and NSE concentration in serum and cerebrospinal fluid in patients with meningitis and meningoencephalitis.
4. Comparison of S100B protein and NSE concentration in patients who presented with neurological and/or psychiatric sequelae one month after recovery and patients with encephalitis yet without sequelae.

## 2 MATERIALS AND METHODS

The concentration of S100B and NSE was based on serum and CSF of 43 patients with TBE treated in the Department of Infectious Diseases and Neuroinfections of the Medical University of Bialystok between 2013 and 2016.

None of the patients had been vaccinated against TBE. Disease was diagnosed based on the clinical picture, presence of inflammatory parameters in the CSF (Table 1), and specific antibodies in serum and CSF according to the case definition that is: the presence of clinical signs of meningitis, meningoencephalitis or meningoencephalomyelitis, an epidemiological link, CSF pleocytosis (>5 cells/dl), and demonstration of recent TBEV infection by the presence of specific serum IgM and IgG antibodies (Taba et al., 2017).

**Table 1:** Comparison of evaluated parameters in the examined groups

Group	*n*	Demographics			Serum						CSF						
		Age	Female	Male	NSE1	NSE2	NSE2/NSE1	S100B1	S100B2	S100B2/S100B1	NSE1	NSE2	NSE2/NSE1	S100B1	S100B2	S100B2/S100B1	
I Meningoencephalitis	17	54.63 ± 13.18	5	12	17.5 ± 18.1	20.7 ± 15	2 ± 2	141.1 ± 80.2	51.4 ± 50.8	0.3 ± 0.3	12 ± 4.8	16.4 ± 7.9	1.6 ± 0.9	186.3 ± 47.5	188.6 ± 67.8	1 ± 0.5	
Ia full recovery	11	53.9 ± 9.5	4	7	13.3 ± 5.2	34.3 ± 9.7	3.6 ± 0.9	148.5 ± 84.6	20.9 ± 30.2	0.1 ± 0.2	12.3 ± 5.6	19.3 ± 9.8	1.9 ± 1.3	191.1 ± 47.4	173.5 ± 60	1 ± 0.6	
Ib sequelae	6	52.5 ± 16.3	1	5	20.5 ± 20.6	16.7 ± 15	1.52 ± 2	134.4 ± 72.1	61.6 ± 48.4	0.4 ± 0.2	10.2 ± 5.2	13.0 ± 6.9	1.4 ± 0.7	177.9 ± 55	167.9 ± 59.1	1 ± 0.5	
*p* Ia versus Ib		ns	ns	ns	ns	0.04	0.04	ns	ns	ns	ns	ns	ns	ns	ns	ns	
II Meningitis	26	50.32 ± 12.53	9	17	13.3 ± 10.8	17.7 ± 8.2	1.6 ± 1.7	127.3 ± 43.1	87.6 ± 53.5	0.5 ± 0.4	6.7 ± 6.7	13.6 ± 10	2.9 ± 3	185.6 ± 33.8	195.8 ± 44.8	1 ± 0.5	
CG	13	52.31 ± 15.34	4	9	17.5 ± 6.3			80.8 ± 58.3			6.7 ± 2.2			183.9 ± 19.1			
*p*‐Value I versus II					ns	ns	ns	ns	ns	ns	0.0002	ns	ns	ns	ns	ns	
*p*‐Value I versus CG					ns	ns		ns	ns		0.04	ns		ns	ns		
*p*‐Value II versus CG					ns	ns		ns	ns		ns	ns		ns	ns		

CSF, cerebrospinal fluid; CG, control group.

*p* I versus II—comparison of analyzed parameters between Sample 1 and Sample 2.

*p* I versus CG—comparison of analyzed parameters between Sample 1 and control group.

*p* II versus CG—comparison of analyzed parameters between Sample 2 and control group.

S100B2/S100B1—ratio of S100B in Sample 2 to Sample 1.

NSE2/NSE1—ratio of NSE in Sample 2 to Sample 1.

TBE antibodies titer was measured with Enzygnost Anti‐TBE/FSME Virus [IgG, IgM] Siemens test.

None of the patients reported any neurodegenerative disorder that could affect the results of the study. Patients were examined by specialists: a neurologist and a psychiatrist.

The treatment was symptomatic with analgesics, anti‐inflammatory drugs, and 20% mannitol.

The concentration of S100B and NSE was measured in serum and CSF on admission (sample 1) and after 14 days of treatment (sample 2).

One month after discharge, all the patients had follow‐up examination in the outpatients’ department for potential sequelae presence.

The TBE patients were divided into two groups depending on the clinical course of the disease:

Group I—patients with meningoencephalitis (*n* = 17: 5 women and 12 men aged between 29 and 79 years; mean – 54.63 ± 13.18 years old). Meningoencephalitis was diagnosed on the basis of consciousness disturbances and/or focal neurological symptoms.

The CSF examinations in this group were as follows: on admission—mean pleocytosis 154.2 ± 92.9 cells/µl, mean protein concentration 51.7 ± 33.4 mg/dl, after 14 days of treatment—mean pleocytosis 76.6 ± 30 cells/µl, mean protein concentration 91.9 ± 50.3 mg/dl. The most common neurological symptom in this group was cerebral ataxia (10 patients).

Group II—patients with meningitis (*n* = 26: 9 women, 17 men aged between 27 and 76 years; mean—50.32 ± 12.53 years old). The CSF examinations in this group were as follows: on admission—mean pleocytosis 98.7 ± 58.1 cells/µl, mean protein concentration 54.4 ± 22.5 mg/dl, after 14 days of treatment—mean pleocytosis 62.6 ± 20.5 cells/µl, mean protein concentration 61.8 ± 29 mg/dl.

Patients diagnosed with meningitis did not present any neurological findings.

Additionally, Group I was divided into two subgroups:

Group Ia—patients with full recovery defined as no neurological or psychiatric symptoms during the follow‐up visit one month after discharge (four female, seven male, mean age 53.9 ± 9.5 year).
Group Ib—patients with neurological or psychiatric symptoms during the follow‐up visit one month after discharge (one female, five male, mean age 52.5 ± 16.3 year). These patients presented with tremors (two patients), ataxia (two patients), paresis (one patient), hearing impairment (one patient), and psychiatric disorders (anxiety, hallucinations—one patient).

The control group (CG) consisted of 13 patients (four women, nine men aged between 27 and 76 years; mean—52.31 ± 15.34 years old). These patients were admitted to the hospital because of headache, and the CSF examination excluded inflammatory process (normal pleocytosis and protein concentration). Additionally, these patients had no history of neurodegenerative disorders. Brain MRI images of these patients did not reveal any significant abnormalities.

Concentration of NSE and S100B was measured by ELISA method using commercial kits according to the manufacturer: NSE and S100B Elisa Kit (DRG, Germany). Samples were collected on admission (sample 1) and after 14 days of treatment (sample 2).

Patients voluntarily agreed to participate in the study and gave their written informed consent. The study was approved by the Local Bioethics Committee.

The results were statistically analyzed using STATISTICA 10. Groups were compared using Kruskal–Wallis test, Mann–Whitney *U* test, Wilcoxon Matched Pairs test, Receiver Operating Characteristic Curve (ROC) tests. Correlations were measured with the Spearman rank test. On the basis of statistical analysis, NSE concentration in CSF seemed to be the best parameters for meningitis and meningoencephalitis differentiation. Therefore ROC curve analysis was performed.

## 3 RESULTS

The groups (Group I, Group II, and CG) did not differ significantly as far as age and sex are concerned (Kruskal–Wallis test).

**Figure 1:**
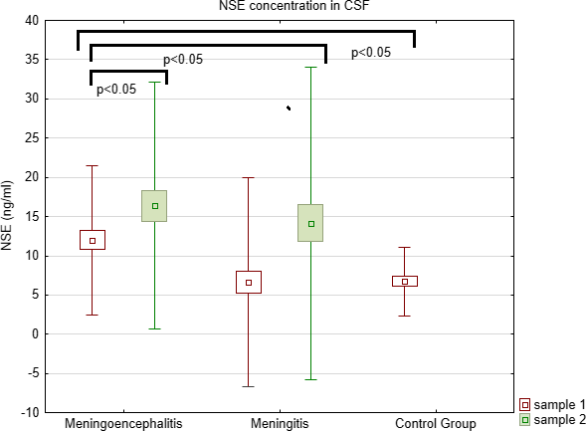
Comparison of NSE concentration in CSF between meningoencephalitis, meningitis, and controls

**Figure 2:**
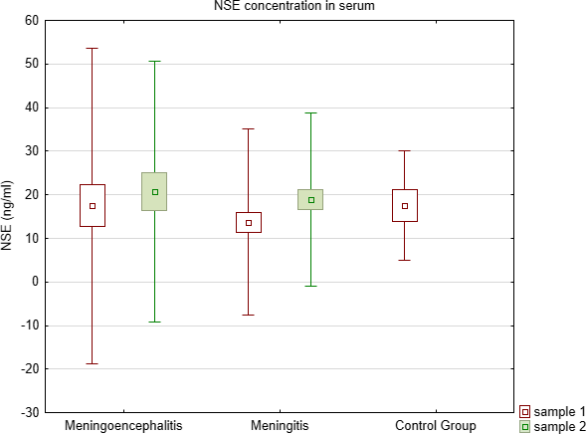
Comparison of NSE concentration in serum between meningoencephalitis, meningitis, and controls

NSE concentration in the CSF was significantly higher (Kruskal–Wallis test) in meningoencephalitis group than in meningitis (*p* = 0.0002) and control (*p* = 0.04) groups (Table 1, Figure 1). There were no significant differences of NSE in serum concentrations (Table 1, Figure 2).

**Figure 3:**
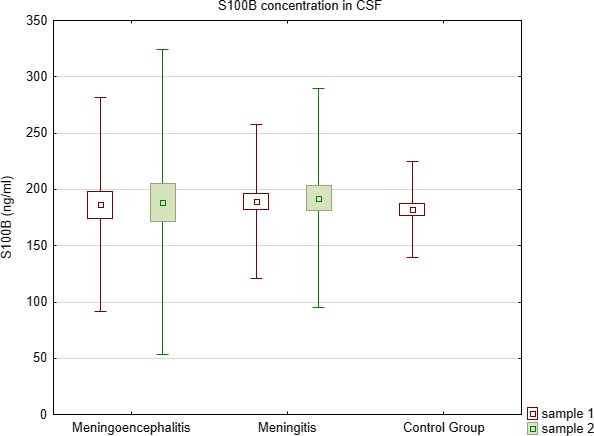
Comparison of S100B concentration in CSF between meningoencephalitis, meningitis, and controls

**Figure 4:**
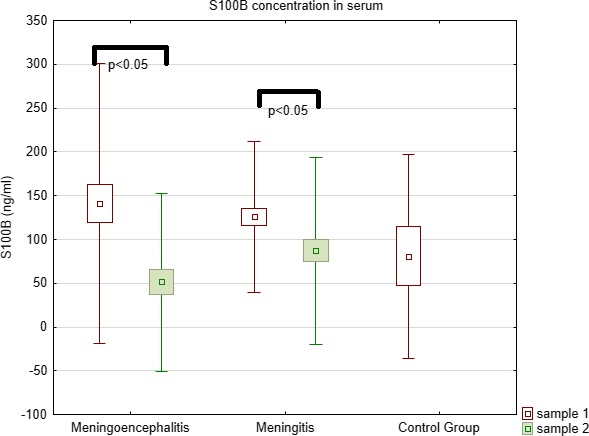
Comparison of S100B concentration in serum between meningoencephalitis, meningitis, and controls

S100B concentrations in both serum and CSF did not differ between all examined groups (Kruskal–Wallis test) (Table 1, Figures 3 and 4).

Comparison of samples (Wilcoxon Matched Pairs test) before and after treatment showed that NSE concentration was higher after treatment in serum and CSF in sample 2 in Group II (*p* = 0.0002 for serum and *p* = 0.002 for CSF). In Group I, a trend toward the increase in CSF and serum was observed, yet the differences were not statistically significant.

Comparison of S100B concentrations (Wilcoxon Matched Pairs test) in both groups before and after treatment (sample 1 vs. sample 2) showed statistically significant decrease in serum (Group I – *p* = 0.006, Group II – *p* = 0.0002).

**Figure 5:**
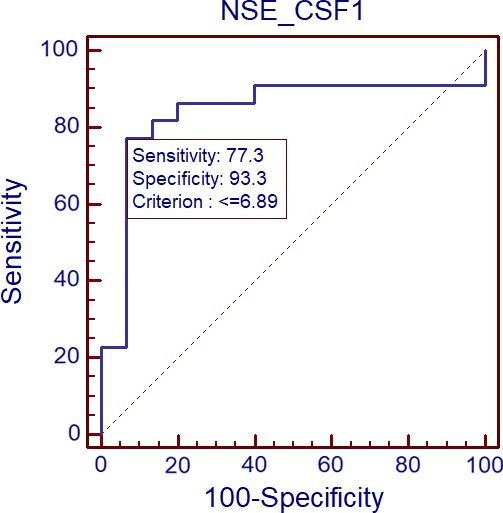
Comparison of NSE concentration in CSF between meningoencephalitis and meningitis groups by the use of ROC plots (*p* = 0.0001). AUC 0.839. Cutoff 6.89

Receiver Operating Characteristic curve analysis indicates that NSE concentration in CSF differentiates meningoencephalitis and meningitis groups (*p* = 0.0001). AUC 0.839. Cutoff 6.89 (Figure 5).

Correlation analysis (Spearman rank test) showed that in TBE patients (both meningoencephalitis and meningitis groups), CSF pleocytosis was significantly correlated with NSE concentration in CSF (*R* = 0.33, *p* < 0.05).

The comparison (Mann–Whitney *U* test) of patients from Group Ia and Group Ib showed that concentration of NSE in the serum sample 2 (after 14 days) was significantly higher in Group Ib (34.3 ± 9.7 vs. 16.7 ± 15, *p* = 0.04) while the concentrations in sample 1 did not differ significantly.

Also, NSE serum sample 2/serum sample 1 ratio was significantly higher (Mann–Whitney *U* test) in the Group Ib than in Group Ia (3.57 ± 0.92 vs. 1.53 ± 1.99, *p* = 0.04).

As far as S100B concentrations are concerned, the statistical analysis (Mann–Whitney U test) showed that serum sample 2/CSF sample 2 ratio was significantly lower in Group Ib than in Group Ia (0.05 ± 0.1 vs. 0.37 ± 0.28, *p* = 0.02).

**Figure 6:**
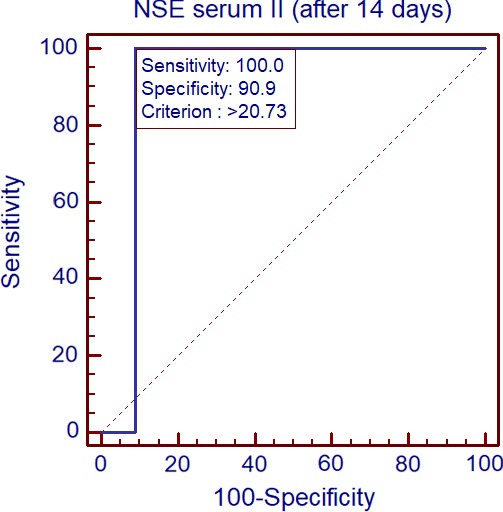
Comparison of NSE concentration in serum II between sequelae group and meningoencephalitis patients by ROC plots (*p* = 0.0001). AUC 0.909. Cutoff 20.73

Receiver Operating Characteristic curve analysis indicates that NSE concentration in serum 2 differentiates Group Ib from Group Ia (*p* = 0.0001). AUC 0.909. Cutoff 20.73 (Figure 6).

**Figure 7:**
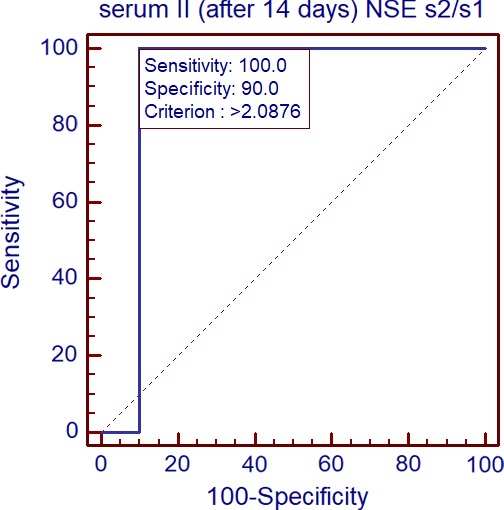
Comparison of NSE concentration ratio serum II/serum I between sequelae group and other meningoencephalitis patients by ROC plots (*p* = 0.0001). AUC 0.900. Cutoff 2.09

Receiver Operating Characteristic curve analysis indicates that NSE concentration ratio serum sample 2/serum sample 1 differentiates Group Ib from Group Ia (*p* = 0.0001). AUC 0.900. Cutoff 2.09 (Figure 7).

## 4 DISCUSSION

Some animal studies suggest that infection with TBE virus may lead to neurodegeneration. Hiranoa et al. reported that alteration in membrane structure and accumulation of TBE viral proteins in dendrites may cause neuronal dysfunction and degeneration in mice. They also demonstrated the hijacking of the neuronal granule system by TBE virus for the transport of viral genomic RNA in dendrites (Hirano et al., 2017).

In our previous study (Czupryna et al., 2016), we observed that patients with a history of TBE in MRI present with cerebral atrophic lesions that cannot be explained by age. This suggested that TBE infection may lead to neurodegeneration.

In our current study, we used S100B and NSE as potential markers of neurodegenerative process during the acute phase of TBE. S100B and NSE were proven to be potential biomarkers of CNS damage (Hajduková et al., 2015).

S100 calcium‐binding protein B (S100B) is a protein of the S‐100 protein family. It is glial‐specific and is expressed primarily by mature astrocytes (Marenholz, Heizmann, & Fritz, 2004; Wang & Bordey, 2008). Its role was assessed in neurological, neoplastic, and other diseases such as Alzheimer's disease, Down's syndrome, epilepsy, amyotrophic lateral sclerosis, schwannoma, melanoma, and type I diabetes (Donato, 2003). S100B has emerged as a candidate peripheral biomarker of blood–brain barrier (BBB) permeability and CNS injury. Elevated S100B levels accurately reflect the presence of neuropathological conditions including traumatic head injury or neurodegenerative diseases. S100B levels have been reported to rise prior to any detectable changes in intracerebral pressure, neuroimaging, and neurological examination findings. The major advantage of using S100B is that elevations in serum or CSF levels provide a sensitive measure for determining CNS injury at the molecular level before gross changes develop, enabling time for medical intervention before irreversible damage occurs.

Serum S100B increases in patients with minor head injury who do not need further neuroradiological evaluation, as studies comparing CT scans and S100B levels have demonstrated, with values below 0.12 ng/ml associated with low risk of obvious neuroradiological changes (such as intracranial hemorrhage or brain swelling) or significant clinical sequelae (Wolf, Ruzicka, & Yuspa, 2010). Studies that focus on traumatic brain injury patients do not show any correlation between a disrupted BBB, using QA, and the peak serum levels of S100B (Bellander et al., 2011), or by using a ratio of the CSF and serum S100B compared to QA (Kleindienst et al., 2010), hence indicating better correlation between actual injury and S100B levels and not the degree of BBB disruption.

Neuron‐specific enolase is a gamma‐homodimer which represents the dominant enolase‐isoenzyme found in neuronal and neuroendocrine tissues. Due to this organ specificity, concentrations of NSE in serum and CSF are often elevated in diseases which result in relatively rapid (hours/days to weeks, rather than months to years) neuronal destruction. Measurement of NSE in serum or CSF can therefore facilitate the differential diagnosis of a variety of neuron‐destructive and neurodegenerative disorders. Its most common application is the differential diagnosis of dementias, such as Creutzfeldt‐Jakob Disease. NSE might be used as a prognostic marker in neuronal injury. Elevated NSE serum concentration correlates with a poor outcome in coma, especially caused by hypoxic insult (Thelin, Nelson, & Bellander, 2017). Several studies have shown that NSE yields a reliable estimate of the severity of neuronal injury as well as clinical outcome of patients with serious clinical manifestations such as in cases of stroke, head injury, anoxic encephalopathy, encephalitis, brain metastasis, and status epilepticus (Lima, Takayanagui, Garcia, & Leite, 2004).

Duda, Krzych, Jędrzejowska‐Szypułka, and Lewin‐Kowalik (2017) reported that there is a significant correlation between mortality in the ICU and increased serum concentration of S100B and NSE, and these parameters might be used as predictors of the treatment outcome in ICU patients.

Our study shows that NSE concentration in CSF of patients with meningoencephalitis is significantly higher than in controls and in patients with meningitis. This confirms that even it is not visible in imaging studies, neurons are damaged in the acute phase of TBE.

The concentration of NSE in the CSF is correlated with lymphocytic pleocytosis.

Other important observation is that NSE and S100B concentrations were increased in sample 2 in meningoencephalitis, which suggests that despite clinical recovery of the majority of patients, the neurons were still being damaged.

Additionally, we selected a group of patients who had not recovered during one month or developed serious neurological or psychiatric sequelae. A comparison of serum sample 2 taken from this group and other patients with meningoencephalitis showed that NSE concentration and serum 2/serum 1 ratio were significantly higher in the sequelae group. It may have practical implication in prediction of TBE course.

In a study performed by Fowler et al., 37 children with TBE were analyzed. The authors observed no differences between children with sequelae and those with a good outcome as far as S100B concentration is concerned. Also, negative correlation between NSE concentration in the CSF and sequelae development was stated. Authors concluded that direct cell damage appears not to play a pivotal role in determining the outcome in TBE during childhood (Fowler et al., 2016).

These results differ from the observations in our study, which most probably results from different clinical course of TBE in children and adults.

Studahl et al. reported that the serum levels of S‐100B in the acute stage of the disease were significantly higher in patients with HSE than in patients with TBE. Also, similarly to our study, the concentrations of S100B in serum of TBE patients did not differ significantly from the control group. The authors explained this with *Herpes simplex* virus cytotoxic effect on glial and neuronal cells, which generates extremely high levels of S100B in the CSF. The protein leaks out in the circulation and can be measured in serum (Studahl, Günther, & Rosengren, 2009).

The limitation of our study was a small number of patients enrolled to the study, especially a very small sequelae group. However, the results are promising and further study will be performed.

## 5 CONCLUSIONS

1. Meningoencephalitis neurodegeneration process is present in TBE.
2. NSE concentration correlates with inflammatory parameters in CSF in TBE.
3. Neurodegeneration is present even after clinical recovery.
4. NSE could be used in prediction of TBE course.

## ETHICAL APPROVAL AND CONSENT TO PARTICIPATE

Patients voluntarily agreed to participate in the study and gave their written informed consent. The study was approved by the Local Bioethics Committee.

## CONFLICT OF INTEREST

Authors declare no conflict of interests.

## AUTHORS’ CONTRIBUTIONS

PC created the idea, applied for money, conducted the study, and prepared the manuscript. SG, SP, KK, JZ, KB,EK collected patients to the study. RS, JD performed laboratory tests. AMM created the idea, helped to apply for money, conducted the study, and prepared the manuscript.
